# Encouraging early outcomes with image guided pencil beam proton therapy for cranio-spinal irradiation: first report from India

**DOI:** 10.1186/s13014-022-02085-4

**Published:** 2022-06-30

**Authors:** Utpal Gaikwad, M. P. Noufal, Jacinthlyn Sylvia, Ashok K. Reddy, Pankaj Kumar Panda, Srinivas Chilukuri, Dayananda Sharma, Rakesh Jalali

**Affiliations:** 1 Department of Radiation Oncology, Apollo Proton Cancer Centre, Chennai, Tamil Nadu India; 2 Department of Medical Physics, Apollo Proton Cancer Centre, Chennai, Tamil Nadu India

**Keywords:** Proton, CSI, Pediatric, Medulloblastoma, Ependymoma

## Abstract

**Background:**

To report our experience with image guided pencil beam proton beam therapy (PBT) for craniospinal irradiation (CSI).

**Materials and Methods:**

Between January 2019 and December 2021, we carried out a detailed audit of the first forty patients treated with PBT. We had recorded acute toxicities, reporting early outcomes and discuss limitations of current contouring guidelines during CSI PBT planning.

**Results:**

Median age of the patient cohort was 8 years, and histologies include 20 medulloblastoma, 7 recurrent ependymoma, 3 pineoblastoma, 3 were germ cell tumors and remaining 7 constituted other diagnoses. Forty percent patients received concurrent chemotherapy. Median CSI dose was 23.4 Gy (Gray; range 21.6–35 Gy). Thirty-five patients (87.5%) completed their CSI without interruption, 5 required hospital admission. No patient had grade 2/ > weight loss during the treatment. Forty-five percent (18) developed grade 1 haematological toxicities and 20% (8) developed grade 2 or 3 toxicities; none had grade 4 toxicities. At median follow up of 12 months, 90% patients are alive of whom 88.9% are having local control. Special consideration with modification in standard contouring used at our institute helped in limiting acute toxicities in paediatric CSI patients.

**Conclusion:**

Our preliminary experience with modern contemporary PBT using pencil beam technology and daily image guidance in a range of tumours suitable for CSI is encouraging. Patients tolerated the treatment well with acceptable acute toxicity and expected short-term survival outcome. In paediatric CSI patients, modification in standard contouring guidelines required to achieve better results with PBT.

**Supplementary Information:**

The online version contains supplementary material available at 10.1186/s13014-022-02085-4.

## Introduction

Craniospinal irradiation (CSI) is an integral component in the standard of care management in a range of CNS tumours; most common is medulloblastoma followed by tumors involving risk of neuraxial spread such as pineoblastoma, GCT, intracranial PNET [1]. Recently in patients with recurrent ependymoma, re-irradiation with CSI and focal boost shown benefit in local control and hence preferred treatment. [2–5]. CSI involves radiation delivery to entire neuraxis, which includes whole brain with meningeal reflections, entire spinal canal and thecal sac. Conventional photon based CSI has been shown to be associated with fair amount of acute toxicities due to large volume of treatment and significant amount of vertebral bone marrow irradiation, causing treatment gaps, risk of infections, hospital admission during treatment particularly with concurrent chemotherapy [6]. Over last six decades, there have been evolution of changes in CSI planning and implementation and current availability of modern proton therapy is emerging as the standard of care whenever available [7, 8]. Proton therapy, by virtue of its physical properties provides dosimetric over modern photon therapy techniques, which are well established [9]. Mahajan et al. in review of CSI using proton therapy reported that, proton therapy was associated with lower acute toxicities and emphasised that further refinement of proton therapy with the use of pencil beam scanning technique might allow further reduction in acute toxicities [8]. In addition, the incorporation of on-board volumetric imaging techniques such as cone beam CT, CT on rails and surface imaging, facilitates daily accurate patient positioning.

We hereby report our institutional experience of CSI using image guided, pencil beam scanning proton therapy in first forty patients, their acute toxicities and early outcomes. In paediatric patients requiring whole vertebral body irradiation, we modified contouring as well as planning approach so as to further limit radiation dose to anterior OARs mainly esophagus, midline mucosa. This approach significantly improved patient compliance to treatment by reducing radiation induced acute toxicities.

## Materials and methods

**Fig. 1:**
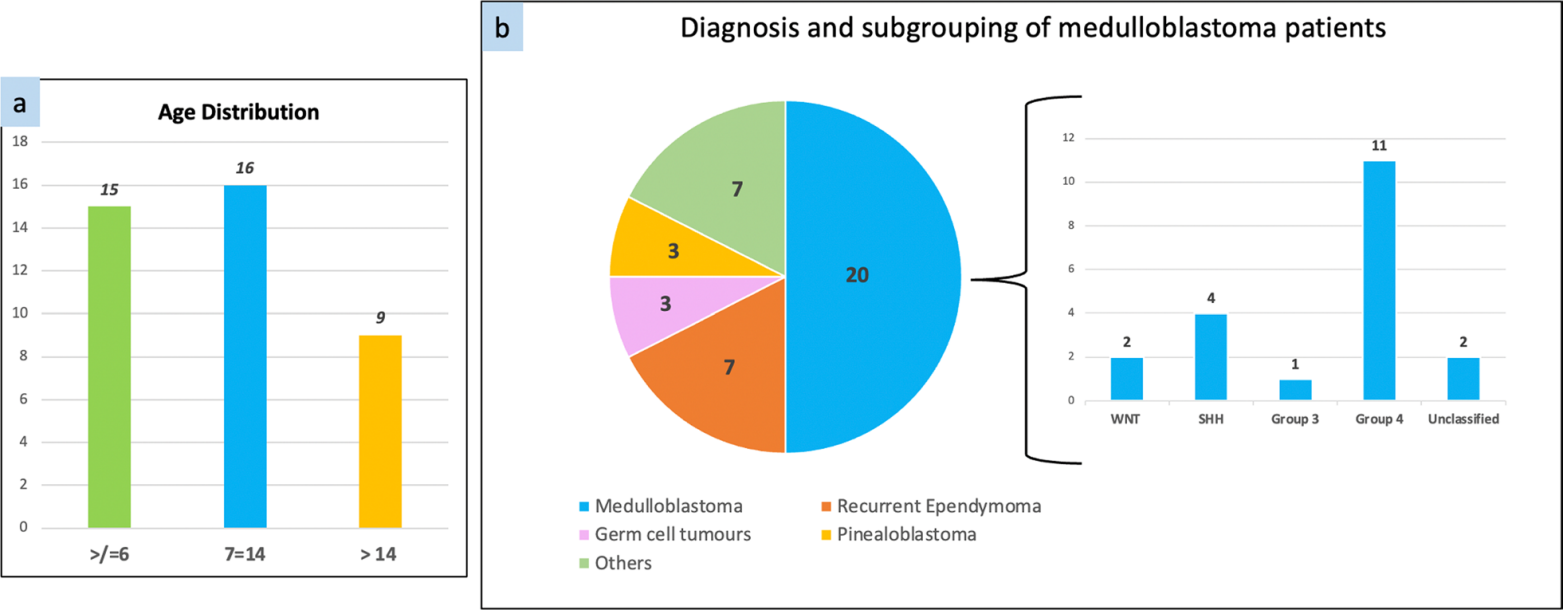
Age distribution (**a**), diagnosis and molecular subgrouping (**b**)

Forty patients with various histologies, who underwent CSI using proton therapy after detailed discussion in the institutional multidisciplinary tumour and proton boards are included in this study. All patients after pre planning audit amongst treating physician, planning physicist and radiation therapist, underwent radiotherapy simulation. A customized neck rest (Moldcare Cushion Qfix, Avondale, USA) and thermoplastic mask (Fiberplast, QFix, Avondale, USA) were used for immobilization of head neck region, with arms by side, in supine position. A customized vacloc either full body or till pelvis, was made for lower body immobilization. Special care taken to avoid any air gap at the junction of head rest and body vacloc; if any gap noticed after planning CT acquisition, it was corrected by repeating immobilisation procedure. Computed Tomography (CT) simulation was performed with 3 mm slice thickness axial images using Canon Aquilion LB CT scanner (Canon Medical Systems, Singapore). Contrast enhanced MRI (Magnetic Resonance Imaging) scan was acquired in treatment position for tumour bed delineation with the aid of pre op MRI scan and planning MRI sequences. For paediatric patients requiring anaesthesia for simulation, immobilisation and planning CT acquisition done by a team of RTT and paediatric anaesthetist who looked after patient during entire treatment, so that immobilisation reproducibility was better and child was comfortable with familiar persons Fig. 1.

**Fig. 2:**
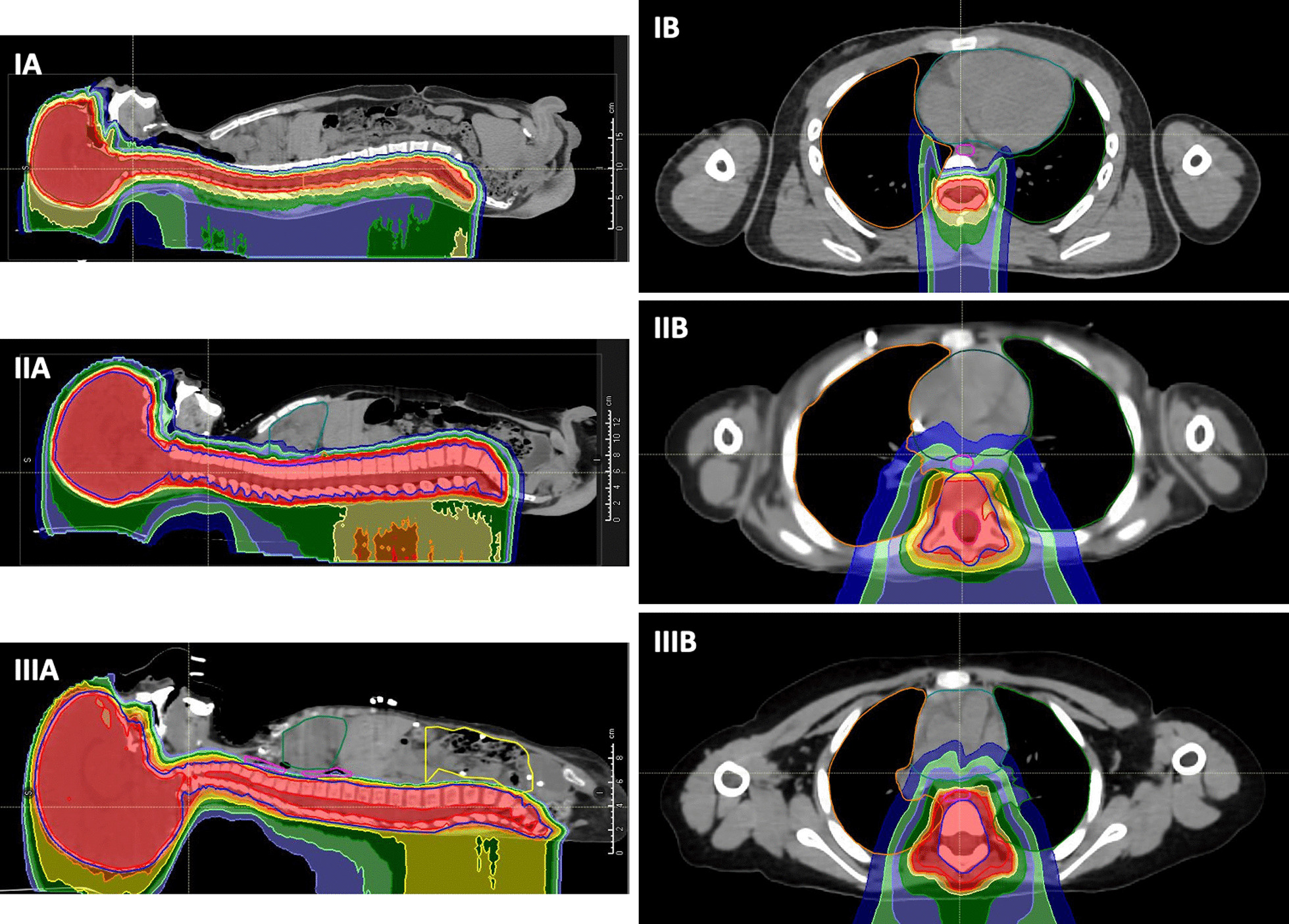
Cranio-spinal dose distribution.**IA** : CSI in young adult, sagittal view. **IB**: CSI in young adult, axial view. **IIA**: CSI in paediatric patient, standard SIOP guidelines, sagittal view. **IIB**: CSI in paediatric patient, standard SIOP guidelines, axial view.**IIIA** : CSI in paediatric patient, modified approach, sagittal view. **IIIB**: CSI in paediatric patient, modified approach, axial view. Colours—Red: Isodose showing 98% of prescribed dose. Orange: Isodose showing 95% of prescribed dose. Yellow: Isodose showing 80% of prescribed dose. Dark Green: Isodose showing 70% of prescribed dose. Light Blue: Isodose showing 50% of prescribed dose. Light Green: Isodose showing 30% of prescribed dose. Dark blue: Isodose showing 10% of prescribed dose

Craniospinal target delineation was done as per standard International Society of Paediatric Oncology (SIOP) guidelines [10]. In adult patients, age more than 14 years; clinical target volume for brain (CTV Brain) delineated which was covering whole brain and dural cuffs of cranial nerves, while CTV spine was covering entire subarachnoid space and nerve roots. Planning target volume (PTV) was generated by expanding CTV brain by 3 mm and CTV spine by 5 mm. For paediatric patients with age less then 14 years, standard contouring guidelines (SIOP and COG) recommends delineation of whole brain with dural extensions as CTV brain, whole vertebral body as spinal CTV. Brain CTV with 3 mm circumferential margin and spinal CTV with 5 mm circumferential margin should be combined to generate CSI PTV [10]. After a detailed discussion among treating physicians, planning physicists and radiotherapy technologists, we modified our approach for paediatric patients; delineated the whole spinal canal with nerve extensions and generated a robust CSI plan with acceptable coverage of whole vertebral body (Fig. 2IIIA, IIIB); no PTV generated for whole vertebral body CTV. This approach was mainly adapted to reduce anterior OAR dose (oesophagus, midline mucosa, thyroid, bowel bag etc.) without significant underdosing or sharp dose gradient in vertebral body region which is known to cause long term skeletal abnormalities. All OARs were drawn and standard dose constraints were prescribed and handed over to physicist to generate CSI plans followed by sequential tumour bed boost plan.

Robustly optimized hybrid proton plans were generated for all patients as per institutional planning protocol using RayStation (Version 9.0, RaySearch Laboratories AB, Stockholm, Sweden) treatment planning system (TPS) [11]. Two posterior oblique fields for brain and one or two posterior beams for spine were used depending on length of the spine. All plans were evaluated for target coverage and OAR dose using standard dose volume indices with robustness of 3 mm translational errors and 3.5% range uncertainties [12]. Robustness for translational errors and range uncertainties was assessed for CTVs, CTV spine in adult patients while CTV spinal canal in paediatric patients.

After radiation plan finalization, and patient specific quality assurance (PSQA) of finalized plan as per institutional protocol [12, 13], all patients received CSI and sequential tumour bed boost. Treatment delivery machine was, Proteus plus which is an isochronous cyclotron based 3 room (2 gantry and 1 fix-beam-line) image guided proton therapy system, equipped with dedicated PBS-nozzle. We can modulate proton energies from 70.2 to 226.2 MeV (Range 4.1–32 g/cm^2^) with corresponding spot size of 6.7–3 mm sigma in air and at isocenter [12].

Daily plan implementation was done after patient position verification using on board CBCT and kilovoltage (KV) x rays under supervision of treating physician and planning physicist. We formulated standard operating procedure (SOP) for imaging, image verification and treatment delivery of CSI after detailed discussion amongst physician, physicist and RTT (Radio-Therapy-Technician), which was used for all patients. We limited use of CBCT specially for paediatric patients so as to limit radiation exposure. (Detailed SOP is in Additional file 1).

Patients underwent quality assurance CT scans at regular interval either after 5–7 fractions or according to CBCT/ clinical findings. All patients were reviewed at least once weekly during radiation therapy, 4 weeks after completion of treatment and thereafter at 3 monthly intervals. Acute adverse events were documented according to Common Terminology Criteria for adverse Events (CTCAE v5.0). Survival outcomes and late toxicities if any were recorded for all patients with median follow up of 12 months (range 3–34 months).

## Results

Of the 40 patients treated with CSI using proton beam therapy at our institute from January 2019 to December 2021, 33 (83.3%) were age less than 18 years at the time of treatment. Their demographic data, details of diagnosis, molecular subgroups, treatment received are summarized in Table 1 and Fig. 1. Dosimetric data for these patients is summarized in Table 2.

**Table 1:** Demographic and treatment details

Median AGE	8 years (1.5 to 37 years)
Age distribution	0–6 years—15 (37.5%) 7–14 years—16 (40%) > 14 years— (22.5%)
Sex ratio (M:F)	32:8
Histopathology	20 (50.0%)—Medulloblastoma 7 (17.5%)—Recurrent Ependymoma 3 (7.5%)—Pineoblastoma 3 (7.5%)—Germ cell tumors 7 (17.5%)—Others (ATRT, PNET, APL with CNS involvement and neuroblastoma)
Reirradiation	Yes—7 (17.5%) No—33 (82.5%)
Anesthesia required	6 (15%)—For simulation and complete treatment 2 (5%)—For simulation only
Radiation dose delivered	Median dose 23.4 Gy 23.4 Gy—20 (50%) 35 Gy—10 (25%) Other—10 (25%)
Concurrent chemotherapy	Yes—16 (40%) No—24 (60%)
Chemotherapy agents used	Vincristine (81.2%) Carboplatin (18.8%)

**Table 2:** Radiation dose details and acute toxicities

	CSI (Total)	CSI (> 14 years)	CSI (< 14 years)
n	40 (100%)	9 (22.5%)	31 (77.5%)
PTV CSI D95 (Avg)	97.4%	99.2%	96.6%
CTV CSI D98 (Avg)	98.6%	99.1%	97.2%
Lens (D max) Avg	2.96 Gy (1.76%)	2.96 Gy (1.76%)	2.96 Gy (1.76%)
Cornea (D Mean) Avg	1.68 Gy (1.34%)	1.68 Gy (1.34%)	1.68 Gy (1.34%)
Cochlea (D Max) Avg	27.3 Gy (93.5%)	27.3 Gy (93.5%)	27.3 Gy (93.5%)
Parotids (D mean) Avg	10.67 Gy (4.63%)	0.44 Gy (0.01%)	12.6 (37%)
Esophagus (D Max) Avg	20.31 Gy (61.6%)	0.00 (0)	27.7 Gy (84%)
Lung (D mean) Avg	0.65 Gy (0.01%)	1.85 Gy (0.07%)	3.22 Gy (0.09%)
Kidney (D mean) Avg	1.85 Gy (0.08%)	2.05 Gy (0.09%)	7.78 Gy (0.23%)
Heart (D mean) Avg	0	0	0
Bowel Bag (D mean) Avg	0.18 Gy (0.007%)	0.01 Gy (0)	0.21 Gy (0.006%)
Liver (D mean) Avg	0	0	0
Gonads (D mean) Avg	0	0	0
Dermatitis Grade 1 Grade 2 Grade 3	20 (50%) 20 (50%) 0	4 (44.4%) 5 (56.6%) 0	17 (54.8%) 14 (45.2%) 0
Mucositis Grade 1 Grade 2 Grade 3	8 (20%) 2 (5%) 1 (2.5%)	1 (11.1%) 1 (11.1%) 0	7 (22.5%) 2 (6.5%) 1 (3.2%)
Weight loss Grade 1 Grade 2	3 (7.5%) 0	0 0	3 (9.6%) 0
Nausea Grade 1 Grade 2	6 (15%) 0	0 0	6 (19.4%) 0
Anorexia Grade 1 Grade 2	7 (17.5%) 0	1 (11.1%) 0	6 (19.4%) 0
Hospital admissions during treatment	5 (12.5%)	Nil	5 (16.1%)
	Average hospital stay = 2.4 days		
Treatment gap during treatment	3 (7.5%)	Nil	3 (9.6%)
	Average treatment gap = 2 days		

Twenty patients (50%) were diagnosed with medulloblastoma, 7 (17.5%) had recurrent ependymoma, 3 (7.5%) pineoblastoma, 3 (7.5%) were germ cell tumors and remaining 7 (17.5%) constituted other diagnoses; namely atypical teratoid rhabdoid tumour (ATRT), primitive neuroectodermal tumour (PNET), acute pro-myelocytic leukemia (APL) with CNS involvement, high grade neuroepithelial tumour and neuroblastoma (Fig. 1a). Among medulloblastoma group, majority were group 4 (55%) followed by SHH (20%) while WNT and unclassified constituted 10% each and one patient was with group 3 (Fig. 1b).

Of all patients, 8 (20%) required short general anaesthesia (sGA) during radiation simulation and of these 8 patients 6 required anaesthesia during entire radiation treatment. Two patients after simulation, and few fractions of radiotherapy under sGA, were managed to complete radiation without it. Median CSI dose prescribed was 23.4 Gy, 40% received 23.4 Gy and 30% received 35 Gy at 1.67 Gy per fraction. Sixteen patients (40%) received concurrent chemotherapy, of whom 13 received injection vincristine and 3 received injection carboplatin. Of 31 paediatric patients, 15 (48.4%) received concurrent chemotherapy and of patients with age more than 14 years, 1 (11.1%) received concurrent chemotherapy. Seven recurrent ependymoma patients received prior focal radiotherapy and median gap between previous radiation and CSI was 3 years (Range 1–5 years).

All patients tolerated treatment well, and only five (12.5%) patients required hospital admission during treatment, all were age less than 14 years. Acute toxicities were documented as per CTCAE v5.0. Only one patient developed grade 3 mucositis during treatment, who was 6 years old boy and was the first patient receiving whole vertebral body CSI. For this patient 3 days of treatment gap and adaptive planning with contour modification was done, after which he completed treatment well. Other than this, grade 1 mucositis was seen in 8 patients and grade 2 mucositis in 2 patients, all were managed conservatively without any treatment gaps. Twenty (50%) patients developed grade 2 dermatitis while remaining had grade 1 dermatitis, no one had grade 3 or more dermatitis.

No patient in this cohort developed grade 2 or more weight loss during treatment and median weight loss was 4.8% of baseline weight, 3 patients lost weight more than 10% of their baseline, all were paediatric age group receiving concurrent chemotherapy. No patient developed grade 2 or more anorexia, nausea vomiting during treatment, grade 1 anorexia was noted in 8 (20%) patients while 6 (15%) had grade 1 nausea vomiting.

Sixty percent [24] developed grade 1 haematological toxicities, of which 6 had grade 1 decrease in haemoglobin, 6 had grade 1 thrombocytopenia and 12 had grade 1 neutropenia. Three patients developed grade 2 neutropenia and 4 patients developed grade 3 neutropenia. No patient developed grade 2 or 3 thrombocytopenia. Six patients had grade 1 haemoglobin decrease, while only patient had grade 2 haemoglobin decrease. All patients who developed grade 3 neutropenia were age less than 14 years and either received prior radiation or concurrent chemotherapy or both. Three of these 4 patients required hospital admission during the treatment, average hospital stay was 2 days and none of them had prolongation of overall treatment. One patient required hospital admission more than once due to VP (ventriculo-peritoneal) shunt blockade.

**Fig. 3:**
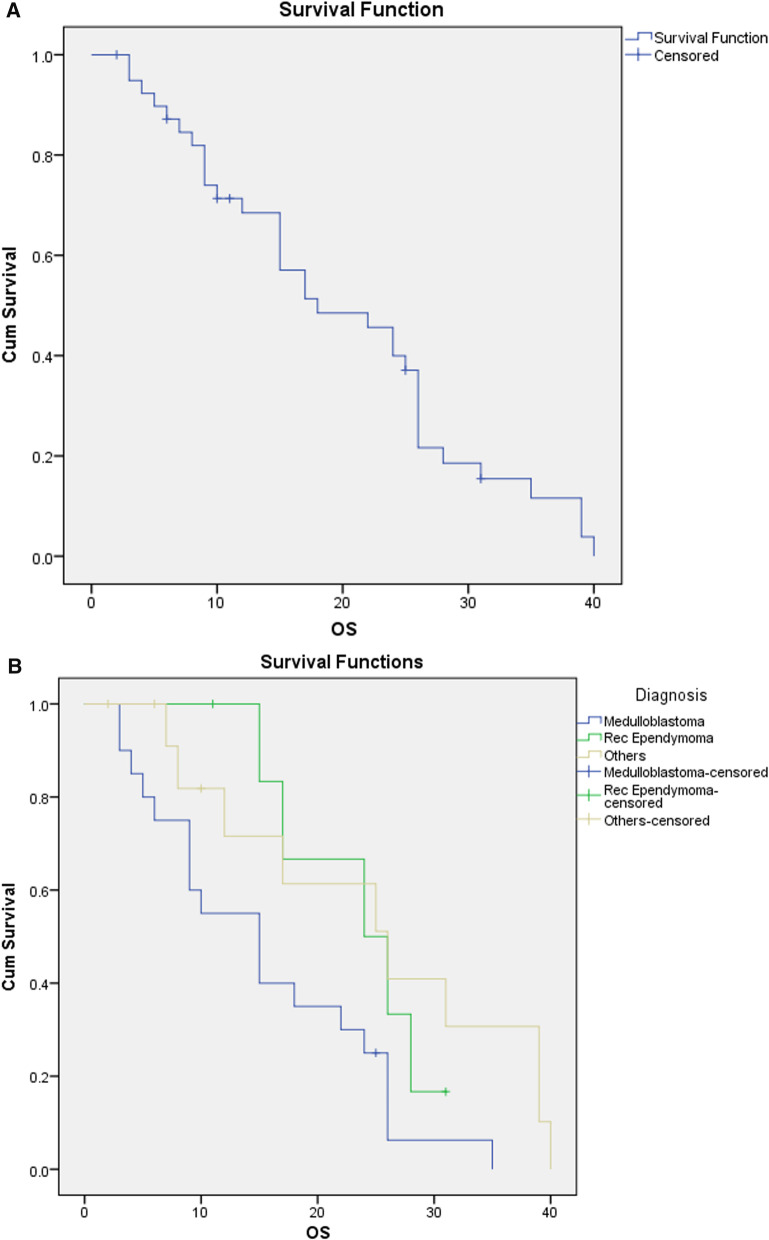
Survival curves. **A** For overall cohort. **B** For Histological subgroups

At median follow up of 12 months (range 3–34 months), 83.3% patients are alive and of them 92% were alive with no evidence of disease (Fig. 3A). We did subgroup analysis, divided patients according to diagnosis in three groups, medulloblastoma, recurrent ependymoma and others and did survival analysis. In medulloblastoma group overall survival was 95%, in recurrent ependymoma group 71.4% while in others it was 76.9% (Fig. 3B).

## Discussion

Benefits of proton therapy across various subsites are well established and its role in low income countries like India is well justifiable [14]. As a first modern proton therapy centre in South East Asia, we treated more than 500 patients over past two years and CNS tumours constituted maximum bulk of it. We have reported our preliminary experience in children and young adults [15, 16].

Indications of CSI and technical evolution in treatment delivery over last few decades we discussed earlier. Delivering radiation to such large volume target is complex procedure and teletherapy machine limitations for such long treatment, warrants use of multiple fields with field junctions. Field junctions, location of vital OARs (Heart, Lungs, Midline mucosa and bowel bag) in close proximity to target and longer survivorship of patients receiving CSI mandates use of highly precise radiation therapy technique.

In addition, most of these patients warranting CSI are of paediatric age group followed by young adults; due all these facts CSI planning and delivery is considered as critical procedure. Over last six decades, CSI techniques evolved tremendously and with availability of proton therapy, it is current standard of care whenever available. In paediatric patients as per current standard recommendations (SIOP and COG) target volume should include whole vertebra so as to avoid or minimize risk of radiation induced skeletal growth abnormalities [10].

Proton beam therapy with pencil beam scanning technique has significantly better dose distribution and OAR sparing when compared with modern intensity modulated radiotherapy and passive scattering proton beam therapy.

In this work, we are analysing acute toxicities of CSI using PBT in both young adult and paediatric patients and their comparison with existing results. Like discussed in various dosimetric studies, proton therapy with no exit dose limits radiation to anteriorly placed OARs due to absence of exit dose [8, 17]. Dose distribution in sagittal and axial sections with OAR sparing achieved for adult patients, paediatric patients with standard approach and our novel approach are shown in Fig. 2. Figure 2 also shows that due to physical properties of proton with no exit dose, it allows limitation of radiation doses to anteriorly placed OARs. In our study population, radiation doses received by all OARs are significantly less when compared with published photon literature and are similar or on lower side compared to proton literature. It is important to achieve good coverage in cribriform plate region as it is one of the common sites of disease recurrence and its proximity to lens and cornea poses difficulty in radiation planning. To achieve better coverage in cribriform region and limit lens, optic apparatus radiation doses we used two posterior oblique fields for brain which in comparison to standard bilateral fields provided more than 98% of prescribed dose coverage without increasing lens dose [11].

Mean dose of bilateral parotids was again significantly less, while average dose received by lungs, kidney and bowel was less than 0.1%. Liver, heart and gonads did not receive any radiation dose. We did subgroup analysis by dividing these patients in paediatric (less than 14 years) and adult age group (> / = 14 years), as all our patients less than 14 years received whole vertebra CSI. In adult patients, radiation dose to all OARs was less than 0.1% of prescribed dose as shown in Table 2. For paediatric patients in whom whole vertebral CSI was planned, we modified standard contouring guidelines as in this group of patients with standard contouring guidelines, vital OARs such as midline mucosa, oesophagus, thyroid gland and bowel bag are partially in PTV volume (as shown in sagittal Fig. 2IIA, and axial section Fig. 2IIB). And this causes either significant dose delivery to the OARs up to 100% of prescribed dose or significant underdosing in the target volume. We mitigated this with our modified contouring method in paediatric patients receiving proton CSI, and like discussed above. We instead of accepting under-dosing or sharp dose gradient in vertebral body region, accepted uniform low dose and verified vertebral body coverage while radiation plan robustness (3 mm translational shifts and 3.5% range uncertainties) was done for CTV including spinal canal and nerve roots. This novel approach, allowed better OAR sparing with no or minimal target underdosing, no dose gradient in vertebral body region.

Dosimetric benefit of proton therapy for CSI is translated in reduction radiation dose to OARs which in turn reduces acute toxicities and improves compliance to treatment. Acute toxicities in our cohort were remarkably less. In overall population most common acute toxicity was dermatitis, 50% patient had grade one dermatitis while remaining 50% developed grade 2 dermatitis and no patient developed grade 3 or more dermatitis. All patients developed dermatitis in scalp region near boost volume and no patient developed grade 2 or more dermatitis in back region near spinal target volume.

Out of these 40 patients, only 8 (20%) developed grade 1 mucositis while 2 (5%) patients developed grade 2 mucositis and one (2.5%) developed grade 3 mucositis. In subgroup analysis, only one patient aged more than 14 years developed grade 1 mucositis, no one developed grade 2 or more mucositis. In patients aged less than 14 years, 22% developed grade 1 mucositis while only two patients (6%) developed grade 2 mucositis and one (3%) developed grade 3 mucositis. Only patient who developed grade 3 mucositis was first paediatric patient receiving whole vertebral body CSI using PBT and contouring was done as per the standard SIOP guidelines (Fig. 2IIA, B). In addition, patient was receiving concurrent weekly injection vincristine. After 5 fractions he developed grade 3 esophagitis, required treatment break, oral opioid analgesics and intravenous fluid administration with supportive care. For him plan adaption was done with partial underdosing of vertebral body in PRV region to limit OAR doses as per Tasson et al. [18]. After this we reviewed existing literature and mitigated this issue with modified approach as discussed above, and with this approach no paediatric patient developed grade 3 or more toxicities.

Haematological toxicities were noticed in 60% of all patients, 15% patients had grade 1 neutropenia, 7.5% had grade 2 neutropenia and only four (10%) patients developed grade 3 neutropenia. Again grade 3 neutropenia was seen only in paediatric patients either receiving chemotherapy or re-irradiation. Only 3 patients developed grade 1 thrombocytopenia, all were receiving concurrent chemotherapy and of these 2 were from paediatric age group. Four patients had decrease in haemoglobin, 3 had grade 1 decrease while one had grade 2; none developed grade 3 anaemia. Gastrointestinal toxicities were noted in 10 patients. No patient in this cohort developed grade 2 or more weight loss during treatment and median weight loss was 4.8% of baseline weight, 3 patients lost weight more than 10% of their baseline, all were paediatric age group. No patient developed grade 2 or more anorexia, nausea vomiting during treatment, grade 1 anorexia was noted in 7 (23.3%) patients while 6 (20%) had grade 1 nausea vomiting.

Hospital admission was required in five patients, all were age less than 10 years, reason for hospital admission was either supportive care, symptomatic treatment or management of neutropenia or VP shunt management. Average hospital stay as we mentioned above was 2 days and this admission during treatment did not prolong overall treatment time for these patients. Three patients, 2 paediatric and one adult patient underwent plan adaptation during treatment, trigger for adaptation was daily CBCT in two patients while one had clinical indication. Reason for plan adaptation were either set up errors due to change in patient characteristics (67%) or acute mucositis (33%).

At median follow up of 12 months, of these 40 patients, 83.3% patients are alive and of them 92% were alive with no evidence of disease (Fig. 3A). We did subgroup analysis, as this cohort had different diagnosis which are strong determinant of their treatment outcomes. We divided patients according to diagnosis in three groups, group one included patients with medulloblastoma, group two included recurrent ependymoma patients receiving CSI as re-irradiation and others were included in third group.

In medulloblastoma group, 19 out of 20 patients are alive with overall survival of 95% at median follow up of 12 months. In recurrent ependymoma group OS was 71.4% while in third group OS was 76.9% (Fig. 3B). When we compared our toxicity and survival outcomes with available proton publications, our patients had comparable or slightly better toxicity profile and similar survival outcomes. Barney et al. in audit of 50 adult CSI patients treated with proton therapy with similar median CSI dose at median follow up of 20 months, noticed median weight loss of 1.6%, grade 1 nausea/vomiting in 46%, grade 2 nausea in 20% and 10% patient had grade 2 or more anorexia, while four patients had grade 3 or more cytopenia [19]. In comparison in our cohort of adult patients, median weight loss was marginally less (1.6% vs. 1.38%), while none had nausea/vomiting, anorexia and cytopenia.

McGovern et al. in their experience of 14 paediatric patients received CSI using PBT, documented grade 3 or more haematological toxicities (Neutropenia, thrombocytopenia and or anaemia) in 50% of patients, while one developed sepsis during the treatment [20]. In comparison, in our cohort only one (4.5%) patient developed grade 3 neutropenia. Considering different histopathologies in both groups survival outcomes are not comparable.

In addition to this toxicity profile, we expect significant risk reduction in late toxicities and risk of second malignant neoplasm in both adult and paediatric patients treated with proton therapy as predicted by various models [21–23], especially in medulloblastoma group where long survivorship is reported [24]. Superior toxicity profile and compliance to treatment in both adult as well as paediatric patients can be attributed to holistic care for each patient, combined team efforts of medical oncologist, dietician, physiotherapist and psychotherapist along with treating physician. We are following standard practice of regular weekly reviews, starting nutritional supplements at the start of treatment and low threshold for intervention. In addition pencil beam scanning technique with daily on board imaging and regular quality assurance CT scan imaging ensured accurate treatment delivery.

Limitations of this study includes a relatively short follow up, mixed age groups and histologies included. We would like to continue follow-up of these patients, document quality of life, late toxicity outcomes and survival outcomes for this cohort.

## Conclusion

We report successful implementation of image guided proton CSI from India. Acute toxicity profile, compliance to treatment and early outcomes of modern proton therapy with pencil beam scanning technique and on board imaging for CSI in both adult and paediatric patients are encouraging. It is important to formulate separate contouring consensus guidelines for whole vertebral body irradiation in paediatric patients, to further extend dosimetric benefits of protons.

## Supplementary Information

**Additional file 1.** Standard operating procedure (SOP) for Imaging and treatment workflow for CSI using protons.

## Data Availability

All data generated or analysed during this study are included in this published article [and its supplementary information files.

## Abbreviations

CSI: Cranio-spinal irradiation
ATRT: Atypical teratoid rhabdoid tumor
PNET: Primitive neuro-ectodermal tumor
APL: Acute promyelocytic leukemia
PTV: Planning target volume
CTV: Clinical target volume
D Max: Maximum dose
D Mean: Mean dose
Avg: Average
