# Supplementary material for: Encouraging early outcomes with image guided pencil beam proton therapy for cranio-spinal irradiation: first report from India
Source: Radiat Oncol. 2022 Jun 30;17:115. doi: 10.1186/s13014-022-02085-4 (PMC9248189; doi:10.1186/s13014-022-02085-4)
Supplement: Supplementary file 1 — Additional file 1. Standard operating procedure (SOP) for Imaging and treatment workflow for CSI using protons. [file 13014_2022_2085_MOESM1_ESM.docx]

**Supplementary file**

**Standard operating procedure (SOP) for Imaging and Treatment workflow for CSI using protons**

Every patient will be aligned and treated by group of three RTTs (radiotherapy technicians), preferably same group for the entire treatment. For patients requiring anesthesia, team of anesthetist, technician and nurse will be present in addition. During first day of implementation, planning physicist and treating physician will be present during patient alignment, image verification and treatment delivery. This process is to make sure the index and anatomy of the patient are reproduced as the simulation day. The index is important because posterior beams going through the couch base hence patient must be indexed to the exact table location for every fraction. The patient’s spine curvature has to be reproduced as in the planning CT.

**Day 1**

Step 1 : Patient verification

Step 2 : Patient alignment at isocenter with the help of laser, using skin markings, markings on head neck mould and vacloc. (These markings are done by simulation team, images of which are captured and uploaded in system; at our institute we have Mosaiq Elekta system). Ensure that the patient is aligned to the sagittal marks from head to lower abdomen. Also make sure the patient is aligned to the vertical marks from chest to hip.

Step 3 : Apply a table shift to the 1^st^ isocenter (ISO). These table shifts are obtained from Mosaiq treatment plan documents uploaded and approved by planning physicist.

Step 4 : Acquire an antero-posterior (AP) and lateral (Lat) kilovoltage (kV) images, match with planning DRR (digital reconstructed radiograph), note the values.

Step 5 : Acquire cone beam CT (CBCT) of brain region; 1^st^ ISO, match images with planning CT and apply the couch correction x, y and z (Use 3D and not 6D—i.e., no rotation, yaw or pitch corrections).

If patient is not aligned to within 3mm, repeat Steps 3 and 4 until the patient is aligned. No need to repeat CBCT so as to minimize radiation exposure unless significant anatomical variation is noted.

- If the couch correction x, y and z (No rotation, yaw or pitch) is no more than 2 mm, don’t apply the shift. Proceed to step 6
- If the couch correction x, y and z (No rotation, yaw and pitch) is more than 3 mm and within 5mm, apply the shift only in **y (longitudinal)**.
- Then, acquire kV images to verify. Repeat ‘kV image 🡪shift couch🡪 kV image’ until the correction is no more than 3 mm. Then, proceed to step 6.
- If the couch correction x, y and z (No rotation, yaw and pitch) is more than 5mm, re-set up the patient and take extra precaution to aligning the patient to the lasers. Then, repeat step 4, verify the correction, if it is within 3 mm.

Step 6 : Save the final couch coordinates for the 1^st^ ISO in Mosaiq Site Setup and record them in the Couch Coordinate Tracking excel sheet manually. Ensure that the coordinates were noted correctly by having an independent 2^nd^ therapist to verify.

Step 7 : Manually shift the couch to the 2^nd^ ISO by applying the treatment plan shift between the two ISOs. The displacement between ISOs should only be in the longitudinal direction. This shift is provided by Physicist in Mosaiq proton plan summary document. The shift is to be verified by two independent therapists.

Step 8 : Acquire an AP and Lat kV images in new couch position

Match with DRR, if the correction is within 3 mm, proceed to Step 8.

If the correction is more than 3 mm, move the patient manually while without moving the couch to correct for the difference. Acquire kV images and verify the correction. If correction is acceptable, proceed to step 8; if not repeat step 7.

Step 9 : Save the final couch coordinates for the 1^st^ ISO in Mosaiq and record them in the Couch coordinate tracking excel sheet. Ensure that the coordinates were inputted correctly by having an independent, 2^nd^ therapist verify.

Step 10: In Mosaiq update the couch coordinates for each iso including treatment fields and setup fields. Ensure that the coordinates were inputted correctly by having an independent, 2^nd^ therapist verify.

Step 11: For patients with 3^rd^ ISO (Longer length of CSI), repeat step 7-9.

Step 12: Record the final table position for the 3^rd^ ISO in the Couch Coordinate Tracking excel sheet

Step 13: Treat the 3^rd^ ISO.

Step 14: ‘Go to’ ISO2, acquire kV images, verify. The couch correction should be no more than 2 mm. If the couch correction becomes larger than 2mm except Y, apply X and Z,

Step 15: Record the final table position for the 2^nd^ ISO in the Couch Coordinate Tracking excel sheet

Step 16: Treat the 2^nd^ ISO.

Step 17: ‘Go to’ ISO1, acquire kV images, verify. The couch correction should be no more than 2 mm. If the couch correction becomes larger than 2mm except Y, apply X and Z,

Step 18: Record the final table position for the 1^st^ ISO in the Couch Coordinate Tracking excel sheet

Step 19: Treat the 1^st^ ISO (brain).

If patient moves in between treatment delivery, repeat kv imaging prior to next step; if shifts are more than 3 mm compared to noted shifts, realign patient and repeat the procedure. Constantly monitor patient with the help of two in room cameras during the entire process.

**Subsequent treatment days**

- Repeat Step 1 to 10 are the imaging process for the whole body. Skip step 4 (CBCT acquisition on daily basis; acquire CBCT twice weekly for adult patients and once weekly for pediatric patients so as to limit radiation exposure)
- Omit image acquisition between two isocenter treatment from day two onwards (Step 14, 17).
- Treat from Iso 3^rd^ or 2^nd^ then move to proximal ones. (This approach of imaging from head to lower body and treatment from lower body to head, minimizes chances of patient movement during treatment)
